# Maternal melatonin levels and temporal dietary intake: results from MY-CARE cohort study

**DOI:** 10.1186/s12884-023-05796-y

**Published:** 2023-07-04

**Authors:** Ai Ni Teoh, Satvinder Kaur, Siti Raihanah Shafie, Nurul Husna Mohd Shukri, Normina Ahmad Bustami, Masaki Takahashi, Shigenobu Shibata

**Affiliations:** 1 Faculty of Applied Sciences, UCSI University, Cheras, Kuala Lumpur, Malaysia; 2 Department of Nutrition, Faculty of Medicine and Health Sciences, Universiti Putra Malaysia, Seri Kembangan, Malaysia; 3 School of Healthy Aging, Medical Aesthetics and Regenerative Medicine, Faculty of Medicine and Health Sciences, UCSI University, Cheras, Kuala Lumpur, Malaysia; 4 Institute for Liberal Arts, Tokyo Institute of Technology, Tokyo, Japan; 5 Department of Electrical Engineering and Biosciences, School of Advanced Engineering and Sciences, Waseda University, Waseda, Japan; 6 Graduate School of Biomedical and Health Sciences, Hiroshima University, Hiroshima, Japan

**Keywords:** Circadian rhythm, Melatonin, Meal timing, Eating behaviour, Pregnancy, Energy distribution, Macronutrient distribution, Chrononutrition

## Abstract

Chrononutrition emerges as a novel approach to promote circadian alignment and metabolic health by means of time-of-the-day dietary intake. However, the relationship between maternal circadian rhythm and temporal dietary intake during pregnancy remains understudied. This study aimed to determine the change in melatonin levels in pregnant women across gestation and its association with temporal energy and macronutrient intake. This was a prospective cohort involving 70 healthy primigravidas. During the second and third trimesters, pregnant women provided salivary samples collected at 9:00, 15:00, 21:00, and 3:00 h over a 24 h day for melatonin assay. Data on chrononutrition characteristics were collected using a 3-day food record. Parameters derived from melatonin measurements including mean, amplitude, maximal level, area under the curve with respect to increase (AUC_I_), and area under the curve with respect to ground (AUC_G_) were computed. A rhythmic melatonin secretion over the day that remained stable across trimesters was observed among the pregnant women. There was no significant elevation in salivary melatonin levels as pregnancy advanced. In the second trimester, higher energy intake during 12:00–15:59 h and 19:00–06:59 h predicted a steeper melatonin AUC_I_ (β=-0.32, p = 0.034) and higher AUC_G_ (β = 0.26, p = 0.042), respectively. Macronutrient intake within 12:00–15:59 h was negatively associated with mean melatonin (Fat: β=-0.28, p = 0.041) and AUC_G_ (Carbohydrate: β=-0.37, p = 0.003; Protein: β=-0.27, p = 0.036; Fat: β=-0.32, p = 0.014). As pregnant women progressed from the second to the third trimester, a flatter AUC_I_ was associated with a reduced carbohydrate intake during 12:00–15:59 h (β=-0.40, p = 0.026). No significant association was detected during the third trimester. Our findings show that higher energy and macronutrient intakes particularly during 12:00–15:59 h and 19:00–06:59 h are associated with the disparities in maternal melatonin levels. Findings suggest the potential of time-based dietary approaches to entrain circadian rhythm in pregnant women.

**Supplementary Information:**

The online version contains supplementary material available at 10.1186/s12884-023-05796-y.

## Background

Circadian rhythm functions as an internal body clock with a periodicity of approximately 24 h. It plays an important role by directing behavioural and physiological activities with respect to the external light-dark cycles, promoting circadian alignment [1]. In circadian rhythm research, melatonin secretion is commonly examined as it is considered the most prominent marker of the circadian rhythm given its role as a circadian messenger and its circadian secretion pattern. Melatonin acts by relaying temporal information signalled from the light/dark cycle from the central clock to the peripheral clocks, stimulating a series of endocrine changes to sync with the circadian rhythm (Gamble et al., 2014). It also has antioxidative properties, making it an important molecule in improving health outcomes [2]. The role of melatonin spans many functions, such as regulating and promoting sleep and reducing risks of depression and obesity by the maintenance of body weight [3, 4].

Viewing the positive role melatonin has on various health outcomes, the exploration into its role during gestation offers new insights into understanding the human programming period. Maternal melatonin can cross the placental barrier freely, elucidating its role in fetal circadian rhythm. In human studies, it was shown that maternal melatonin level influences fetal melatonin secretion in a similar pattern, and whenever the maternal rhythm is disrupted, the same disruption can be observed in fetal melatonin patterns [1, 5]. Disruption in maternal circadian rhythms has been associated with a significant difference in fetal number and reduced fetal weight [6], lower pregnancy success rate [7], extended gestation length [8], and postnatal behavioural circadian rhythms in the offspring [9]. Early studies done on mammalians showed that maternal circadian rhythm entrains fetal/infant rhythm from pregnancy to near birth by showing evidence of higher glucose utilization in the day as compared to night from embryonic activity [10–12].

The suprachiasmatic nucleus (SCN) regulates the temperature and feeding in mammals which are entrained to hormonal rhythms. One major influence of this entrainment is the light/dark cycle which ultimately influences the peripheral clock that regulates nutrient metabolism through food intake [13]. However, meal timing too has a potent role to play in the regulation of peripheral clocks. Research in the area of circadian entrainment in relation to diet suggests that chrononutrition or the study of meal timing for circadian alignment serves as important nutrient-related and temporal cues to the circadian system, particularly at the peripheral level [14]. Chrononutrition can entrain the peripheral clocks through signals generated upon feeding and fasting, such as hormones, metabolites, post-prandial temperature rise, and intracellular redox processes [15–17]. This is also attributed to the circadian nature of the feeding/fasting cycle, where energy is replenished during the diurnal active phase and mobilization of energy stores takes place during the resting phase. Aligning the timing of food intake with the daily active/resting phase is thought to exhibit a greater metabolic benefit because the physiological systems are more ready for the nutritional challenge during this period. Evidence from both animal and human studies showed that mistimed food intake can reset the phase of rhythmic gene expression in peripheral organs, leading to circadian disruption [18–21]. There is a cumulative body of research that suggests that some characteristics of chrononutrition, such as time-restricted feeding [22], breakfast-skipping [23–25], night eating syndrome (NES) [26], and late-night eating [27–29] can potentially alter circadian rhythm [22, 23]. Taken together, these data suggest the entrainment effect of temporal dietary patterns on the circadian rhythm that may potentially confer a greater disruptive effect on health.

While previous studies on maternal dietary intake largely focused on the quantity and quality of dietary intake, the temporal aspect of maternal dietary intake remains understudied. As circadian alignment is largely associated with optimal maternal and pregnancy outcomes, determining the role of chrononutrition on maternal circadian rhythm would provide useful insights into potentially modifiable eating behaviours that favour an optimal circadian rhythm. Identifying the dietary factors associated with maternal circadian rhythm during pregnancy is practically imperative for designing targeted preventive care and interventions to promote a healthy pregnancy, which can benefit both the mother and the child. Hence, this study aimed (1) to determine maternal melatonin rhythm during the second and third trimesters; (2) to examine the temporal distribution of energy and macronutrient intakes of pregnant women during the second and third trimesters; (3) to determine the association of maternal melatonin rhythm with temporal energy and macronutrient distribution during the second and third trimesters. It is hypothesized that energy and macronutrient intakes are associated with aberrant melatonin secretion during the second and third trimesters of pregnancy in a temporal manner.

## Methods

### Study design and data collection

Data described in this paper were derived from a larger MY-CARE observational cohort study conducted to determine maternal circadian rhythm during gestation and its association with birth outcomes and infant growth [30]. The study was conducted from June 2019 to October 2021. Healthy primigravidas with singleton pregnancy, aged between 19 and 39 years, and in their first 20 weeks of gestation were invited to participate in the study. Subject recruitment was carried out at ten randomly selected government maternal and child clinics (*Klinik Kesihatan Ibu dan Anak*) in Kuala Lumpur, Malaysia. The exclusion criteria were: pre-existing health complications such as diabetes mellitus, hypertension, and anaemia, pregnancy-related complications, physical disabilities, intake of medicine or supplement containing melatonin or corticosteroids, consumption of sleep medicine, usage of recreational drugs, smoking, involvement in shift work, or undertook transmeridian flight in the past 3 months at recruitment.

The study’s required sample size was computed using G*POWER software, version 3.1.9.4 (http://www.gpower.hhu.de). Using a linear multiple regression method, effect size (f^2^) of 0.338 [31], type I error rate (α) of 0.05 and power (1-β) of 90%, the minimum sample size needed was 49. Considering 20% of drop-out and non-compliance, the targeted sample size was increased to 60.

A total of 138 eligible primigravidas gave their consent and agreed to participate upon recruitment at the clinics. Throughout the study, a total of 51 participants withdrew from the study due to various reasons as depicted in Additional File 1. There were 17 primigravidas who provided only a single trimester salivary sampling in the second or third trimester. Hence, the final number of primigravidas that were included in the analysis was 70. There was no significant difference in the socio-demographic characteristics between the primigravidas included in this study and those who discontinued the study.

Data on socio-demographic characteristics of the primigravidas, including age, race, educational level, household income level, and health history were collected using a face-to-face questionnaire at the clinic upon recruitment. Pre-pregnancy weight and height were extracted from the antenatal booklet. All participants provided their written informed consent before data collection began. Ethical approval for the study was obtained from the Medical Research and Ethics Committee (KKM/NIHSEC/P19-125) prior to the commencement of the study.

### Temporal dietary intake

A 3-day food record was used to determine the dietary intake of pregnant women during their second and third trimesters. Information on the timing of meal or snack, occasion, details of food, beverage, and supplement, and the amount consumed were recorded. The 3-day food record required the records on 3 non-consecutive days within a week, consisting of two weekdays and one weekend day. The pregnant women were encouraged to record as many details of the food and beverage as possible, such as the cooking method and the source of cooked or ready-to-eat meals. Household measurements, including cups, plates, bowls, teaspoons, and tablespoons were used in the estimation of portion size. To minimize recall bias, subjects were advised to self-record their dietary intake as soon as they finished their meal. A review was conducted with the pregnant women upon returning the 3-day food record to ensure no information was missed.

For data analysis, the amount or quantity of food and beverages reported by the pregnant women were converted into metric units (gram or millilitre). The mean energy and macronutrient intakes were determined using the NutritionistPro® software (Axxya System, 2008). Malaysian Food Composition Table [32] and USDA Standard Reference Database [33] were referred to for the dietary analysis. For food items not found in the Nutritionist Pro, ASEAN Food Composition Table [34] and Singapore Food Composition Guide [35] were used as guidelines. The mean energy and macronutrient intake were compared with the Recommended Nutrient Intake (RNI) for Malaysians to determine intake adequacy while considering the additional energy requirements of 280 kcal/day for the second trimester and 470 kcal/day for the third trimester of pregnancy [36]. To identify under-reporting of energy intake, the ratio between reported total energy intake (EI) and basal metabolic rate (BMR) was computed, whereby a ratio of < 1.2 was regarded as under-reporting whereas a ratio of > 2.4 was categorized as energy over-reporting [37, 38].

Temporal distribution of percent total daily energy intake (%TDEI) and percent macronutrients (carbohydrate, protein, and fat) intake by TDEI was calculated. Malaysia is located close to the equator, with the Latitude of 1˚ and 7˚ North and Longitudes of 100˚ and 119˚ East Malaysia [39]. In Malaysia, sunrise and sunset occur at approximately 7:00 h and 19:00 h throughout the year, with ~ 12 h of daylight throughout the year [40]. Taking into consideration the biological day and night timings, the temporal distribution by 24 h day for this study was divided as followed: 7:00 to 11:59 h, 12:00 to 15:59 h, 16:00 to 18:59 h, 19:00 to 23:59 h, and lastly 24:00 to 06:00 h, in reference to a previous study conducted in Singapore [41]. The time interval corresponds to the typical meal intake pattern over the day to capture the circadian consumption of breakfast, lunch, dinner, and snacks, while providing information on temporal eating by the biological day and night.

### Salivary melatonin levels

Similarly, during the second and third trimesters, all pregnant women provided salivary samples. The passive drool method was used to collect whole salivary samples at 9:00, 15:00, 21:00, and 3:00 h on a work-free day when the pregnant women were able to follow their preferred sleep/wake timing and usual routines. Pregnant women were advised to refrain from consuming chocolate, banana, alcohol, caffeine and drinks containing artificial colourants to prevent the contamination of salivary samples with compounds that can interfere with the assay [42–44]. Nicotine and prescribed over-the-counter medicines were required to be documented and reported to the researcher. Pregnant women were also required to report their wake and sleep time on the sampling day. Pregnant women were instructed to collect 3.0 mL of salivary sample in an aluminium foil-covered centrifuge tube through a funnel. The salivary samples were kept in the home freezer immediately after collection. Upon returning to the lab, salivary samples were centrifuged at 3,000 g at 20 °C for 10 min to collect supernatants. Subsequently, the samples were stored at -20 °C until further analysis. Analysis of salivary melatonin concentration started by homogenizing and centrifuging the thawed samples at 10,000 g for 5 min at room temperature. Salivary melatonin concentration was determined using direct melatonin direct saliva ELISA kit produced by IBL International (Hamburg, Germany) which has an analytical sensitivity of 0.3 pg/ml and an assay range of 0.5 to 50 pg/ml. Samples containing melatonin concentrations that exceeded the standard range of the ELISA kit were diluted and re-assayed. All samples from each participant were analysed in a single assay. Measurements were made in duplicate and converted from pg/ml to pmol/l. The intra- and inter-assay coefficients of variation were 2.4% and 13.0% respectively.

### Melatonin and potential covariates

Study design-related factors (gestation week at sampling), maternal socio-demographic characteristics (maternal age, race, household income level, educational level, and pre-pregnancy body mass index (BMI)), situational factors (wake and sleep time on sampling day and sleep duration), and fetal sex were identified as potential covariates that may influence melatonin secretion. Hence, preliminary analyses were conducted using one-way ANOVA, independent sample t-test, and Spearman’s correlation test to examine the univariate associations between the abovementioned variables and melatonin parameters. Variables that showed significant association with melatonin levels were included as controls in the subsequent analyses.

### Statistical analysis

All statistical analysis was conducted using SPSS software version 20 (SPSS Inc., Chicago, IL, USA). Continuous data with a normal distribution were reported as mean and standard deviation (SD) whereas median and interquartile range (IQR) were reported for skewed data. Categorical variables were reported in percentage. The Shapiro Wilk test was used to check for outliers and skewness in the distribution of data. Samples outside ± 3 SD from the mean were considered outliers and excluded from the analysis. A *p-*value of < 0.05 was considered statistically significant. The differences between the variables in the second and third trimesters were tested using paired sample t-test (for parametric variables) and Wilcoxon’s sign rank test (for non-parametric variables). Preliminary analyses using Spearman’s rank correlation test were performed to determine the significant covariates of melatonin levels.

The amplitude of melatonin levels across the day was calculated as the ratio of the highest value to the lowest value of melatonin measurements within the 24 h day [45]. The maximal melatonin level refers to the highest value among the four melatonin measurements. The area under the curve with respect to ground (AUC_G_) and the area under the curve with respect to increase (AUC_I_) were computed using the trapezoid method [46]. The AUC_G_ represents the total output of melatonin levels over the 24 h day. The AUC_I_ represents the degree of increment in melatonin concentrations over the day, where a higher value indicates a steeper increment of melatonin across the day. The change in melatonin parameters and dietary intake from the second to the third trimester was computed by deducting the measurements in the second trimester from that in the third trimester. This was done to evaluate the relationship between melatonin parameters and dietary variables in terms of changes over the trimester.

Hierarchical linear mixed modelling (LMM) was used to evaluate the effect of time and trimester on maternal melatonin concentrations over the day. This statistical approach is useful to examine nested data and to account for between-person and within-person variations. The distribution of absolute melatonin concentrations at all the time points remained positively skewed after the removal of outliers, hence log-transformed values were used for the mixed model analysis. Firstly, a full-factorial model including sampling time and trimester with a random intercept and a random effect of time was carried out to allow individual variation in the effect of time on melatonin concentrations. The time of sampling was centred at 9:00 h. The changes in melatonin concentrations in the morning (9:00 to 15:00 h) and evening (15:00 to 03:00 h) were examined separately, considering the nature of melatonin levels that typically reaches the nadir in the middle of the day. A quadratic effect of time was also entered into the model to determine the change in the steepness of melatonin levels over time. The interaction between sampling time and trimester was entered into the model to determine the change in melatonin concentration across trimesters. The addition of random effect was tested using the likelihood deviance difference test (α = 0.05) using restricted maximum likelihood (REML). Next, covariates of melatonin levels that were identified in the preliminary analyses were included as controls in the LMM. Models were fitted with maximum likelihood (ML) for the interpretation of fixed effects. Model selection was based on the Aikake Information Criterion (AIC) and tested with likelihood deviance difference test (α = 0.05). The coefficients in LMM were reported as percent change in outcome per unit change in the independent variables using the equation β_%change_ = [exp(βln)]– 1 [47].

To examine the association between maternal melatonin levels and temporal dietary intake variables, hierarchical linear regression analyses with the enter method were performed while adjusting for covariates. The potential covariates identified from the preliminary analyses were entered in the first step, whereas the temporal dietary intake variables were entered in the second step of the hierarchical regression model to allow the examination of their incremental predictive effect on the melatonin variables. Maternal age and gestational weight at sampling were entered in the first step to account for between-subject variation, followed by pre-pregnancy BMI, sleep time, and fetal sex which were identified as significant covariates in the preliminary analyses. Firstly, R squared change and the p-value of the chrononutrition variables were evaluated to determine the significance of the value in explaining the additional variance of the model. Secondly, the F value of the overall model was examined to determine the significance of the overall model, including the covariates, in predicting the melatonin variables. Collinearity between variables entered in the model was tested with variance inflation factors (VIFs), where VIF greater than 5 indicates collinearity.

## Results

### Subject characteristics

The mean age of the pregnant women in the present study was 18.7 years. Out of the 70 pregnant women, the majority (55.7%) were Malay and had tertiary education (84.3%). A higher proportion of the pregnant women had a middle household income level (47.1%) or a high household income level (42.9%). In terms of pre-pregnancy BMI, 10.0% were underweight, whereas 23.4% were overweight or obese before pregnancy. The mean gestational week of the pregnant women upon data collection was 20.0 (range from 13 to 27 weeks) during their second trimester and 32.9 weeks (range from 28 to 37 weeks) during their third trimester. The subject characteristics were summarized in Table 1.

**Table 1:** Characteristics of the study sample (n = 70)

	Mean	SD	Range	n	%
**Age (years)**	28.7	3.7	20–37		
**Gestational week (weeks)**					
T2	20.0	3.7	13–27		
T3	32.9	2.1	28–37		
**Pre-pregnancy BMI (kg/m** ^**2**^ **)**	23.3	4.9	15.8–42.3		
Underweight				7	10.0
Normal				47	67.1
Overweight				12	17.1
Obese				4	5.7
**Ethnicity**					
Malay				39	55.7
Chinese				26	37.1
Indian				2	2.9
Others				3	4.3
**Educational level**					
Secondary				11	15.7
Tertiary				59	84.3
Household income (RM)*					
Low (< 2300)				7	10.0
Middle (2300–5599)				33	47.1
High (≥ 5600)				30	42.9

Abbreviations: BMI = body mass index; RM = Ringgit Malaysia; T2 = second trimester; T3 = third trimester.

### Melatonin and potential confounding variables

No statistically significant correlation was found between melatonin variables in the second and third trimesters with maternal age, race, gestational week, household income level, education level, wake time, and sleep duration (all p > 0.05). In the third trimester, pre-pregnancy BMI was significantly correlated with melatonin level at 15:00 h (r=-0.426, p = 0.002). Additionally, sleep time on sampling day was negatively correlated with AUC_I_ (r=-0.343, p = 0.010).

Pregnant women who carried a female fetus displayed a significantly steeper melatonin AUC_I_ than those carrying a male fetus in the second trimester (p = 0.033). During the third trimester, mean melatonin level (p = 0.039) and maximal melatonin level (p = 0.043) also significantly varied between fetal sex, where pregnant women carrying a female fetus had significantly higher mean and maximal melatonin levels. Hence, pre-pregnancy BMI, sleep time, and fetal sex were entered as control variables in subsequent regression analyses.

### Circadian variation in salivary melatonin concentrations

**Fig. 1:**
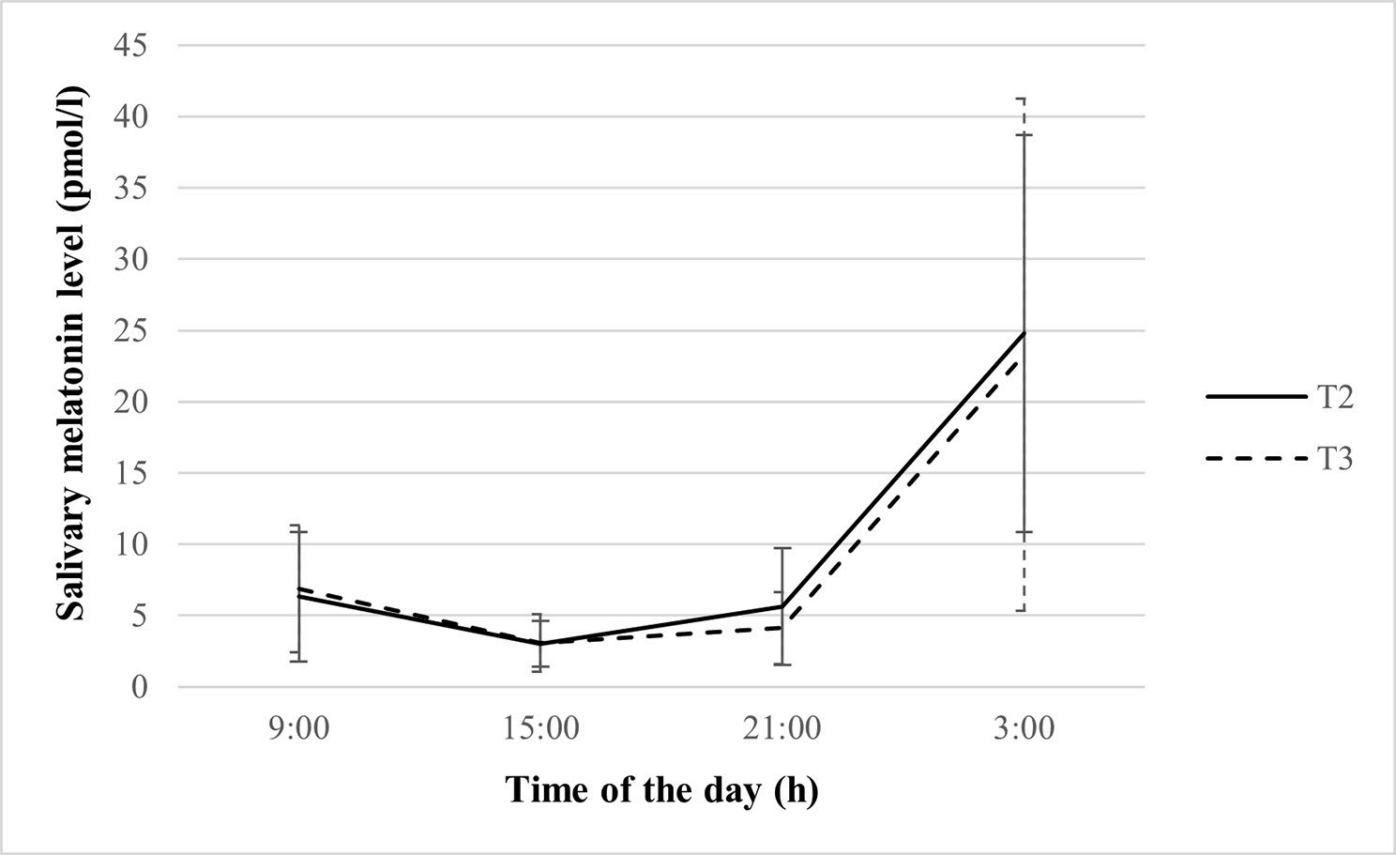
Salivary melatonin levels of pregnant women across trimesters (n = 70). Values are expressed in median ± median absolute deviation. Note. T2 = second trimester; T3 = third trimester

A distinct rhythm of melatonin secretion characterized by a nocturnal increment in melatonin concentration from 21:00 h to 3:00 h was observed among pregnant women across trimesters. Subsequently, the melatonin level decreased substantially from 9:00 h in the morning and reached the lowest value at 15:00 h (See Fig. 1). The results are consistent with previous studies that showed a nocturnal rise in melatonin. A greater variability in the melatonin level across the pregnant women was observed for the sample collected at 3:00 h, as depicted in Fig. 1. This may be attributed to the intrinsic differences in individual melatonin levels due to physiological factors and disparities in the sampling conditions between individuals.

The values of melatonin concentration and parameter were summarized in Additional File 2. The sleep time of pregnant women was significantly later during the third trimester than during the second trimester (p = 0.011). There was no significant difference in melatonin concentrations at 9:00, 15:00, 21:00, and 3:00 h (all p > 0.05). Melatonin parameters including mean, maximal level, AUC_G_, and AUC_I_ were maintained across trimesters (all p > 0.05). However, the amplitude of melatonin levels across the day was significantly lower in the third trimester as compared to the second trimester (p = 0.020).

Results from the linear mixed model in Table 2 showed the changes in melatonin concentrations over time and across trimesters. The addition of a random effect of time did not significantly improve the model fit (deviance difference (2) = 7.50, p = 0.972). However, to allow individual variations in the effect of time on the melatonin level, the random effect of time and random intercept remained in the full model. The full mixed model included the fixed effects of time, trimester, and random effect of time. Results showed that the mean melatonin concentration across the day did not significantly vary between trimesters (p = 0.951). In the morning from 9:00 to 15:00 h, the melatonin concentration was declining at a significant rate of 8.9% per hour. The interaction between time and trimester did not appear to be significant (p = 0.903), suggesting the lack of significant difference in the melatonin slope in the morning across trimesters (p = 0.903). From 15:00 to 3:00 h, melatonin concentration increased at a significant rate of 9.3% per hour. The quadratic effect of time appeared to be significant (p = 0.004), indicating that the rate of increment in melatonin concentration over time was 3.0% steeper per hour. There was no significant variation in the melatonin increment in the evening across trimesters (p = 0.935). The random effects indicated that the intercepts and slopes for the relationship between time and melatonin levels did not vary significantly at the person level.

**Table 2:** Full linear mixed model for melatonin levels across the day (n = 70)

Melatonin model					
Variables	Estimate	S.E.	t-statistic	*p*-value	Interpretation
Intercept	1.819	0.156	11.656	< 0.001**	6.17pmol/l
Trimester = 2	-0.007	0.107	-0.062	0.951	0.7% lower melatonin in T = 3
**Morning (9:00–15:00 h)**					
Collection time (linear)	-0.429	0.079	-5.383	< 0.001**	8.9% decrease per hour
Collection time × Trimester	-0.013	0.108	-0.122	0.903	1.3% steeper in T = 3
**Evening (15:00–03:00 h)**					
Collection time (linear)	0.442	0.035	12.687	< 0.001**	9.3% increase per hour
Collection time (quadratic)	0.163	0.002	2.900	0.004**	3.0% steeper per hour
Collection time × Trimester	0.002	0.011	0.082	0.935	0.2% steeper in T = 3
**Random effects**	**Variance component**		**S.E.**	**Wald z**	***p*** **-value**
Residual	0.23		0.02	11.94	< 0.001**
Intercept (person-level)	0.46		0.27	1.68	0.093
Covariance (person-level)	-0.38		0.24	-1.60	0.109
Time (person-level)	0.36		0.22	1.61	0.107

Abbreviations: S.E. = standard error; T = trimester

*p < 0.05; **p < 0.01; ***p < 0.001

### Temporal energy and macronutrient distribution

The number of chrononutrition data that was included in the final analysis was 64 and 60 for the second and third trimesters after removing underreported data. The mean energy intake of the pregnant women was 2205 ± 413 kcal/day and 2119 ± 304 kcal/day during the second and third trimesters, respectively. This indicates that pregnant women in this study achieved an average of 104.0% and 91.75% of the Recommended Nutrient Intake (RNI) across trimesters [48]. The majority of the pregnant women achieved or exceeded the recommended RNI for protein consumption, with a prevalence of 92.2% and 75% during the second and third trimesters, respectively. In terms of fat intake, a higher proportion (81.3% and 51.7%) exceeded the recommended RNI of 60-71 g/day for the second trimester and 65-78 g/day for the third trimester. Across trimesters, there was no significant difference in the mean energy, carbohydrate, protein, and fat intakes (all p > 0.05).

**Fig. 2:**
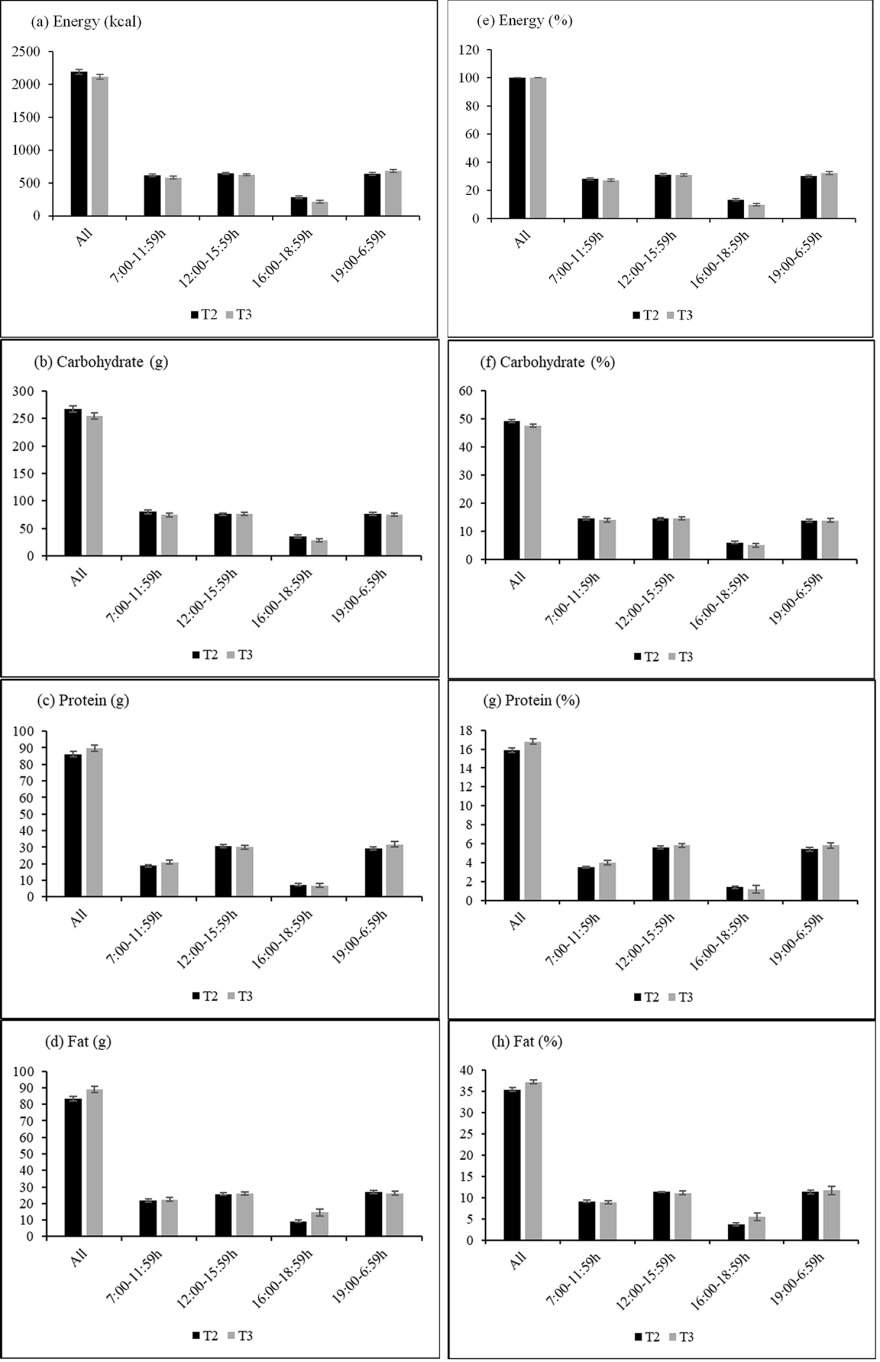
Temporal energy and macronutrient distribution during the second (T2, n = 64) and third trimester (T3, n = 60)

As shown in Fig. 2, energy intake in the afternoon and at night was higher than that in the morning, indicating a delayed temporal energy intake. Night eating or energy intake after midnight was not observed in the present study, with 1% of energy consumed during the period of 24:00 to 6:00 h on average. The temporal distribution of macronutrients by the 24 h day across trimesters was depicted in Fig. 2. Despite the lack of significant difference, it was observed that the carbohydrate intake was slightly higher in the morning (14.7–14.8%) and afternoon meals (14.2–14.5%) than in evening meals (13.2–13.6%). In contrast, a higher proportion of protein was consumed in the afternoon (5.6–5.9%) and at night (5.0-5.7%) than in the morning (3.5–3.9%). Similarly, fat intake was higher in the afternoon (11.0-11.1%) and evening meals (11.4–11.6%), with a lower intake in the morning (8.9-9.0%). The mean intake of energy and macronutrient in g/d across the 24 h day during the second and third trimesters was listed in Additional File 2.

### Association between maternal melatonin levels and temporal energy and macronutrient intake

Table 3 shows the results of the hierarchical linear regression model on the association between maternal melatonin parameters and temporal energy intake. Trimester-specific associations were detected. In the second trimester, a higher %TDEI in the early afternoon (12:00–15:59 h) was significantly associated with a flatter melatonin AUC_I_ (β=-0.32, p = 0.034), explaining an additional 8.4% of the variance. During the night from 19:00 h to the next morning at 6:59 h, a higher %TDEI during this period was associated with a greater melatonin AUC_G_ (β = 0.26, p = 0.042), accounting for an additional 9.7% of the variance.

**Table 3:** Hierarchical linear regression models predicting maternal melatonin parameters from temporal energy intake (n = 70)

	Melatonin parameters during the second trimester			
	AUC_I_		AUC_G_	
	β	*p*-value	β	*p*-value
*Step 1*:				
Maternal age	0.07	0.626	0.37	0.006**
Pre-pregnancy BMI	-0.21	0.140	-0.23	0.076
Gestational age	-0.08	0.535	-0.15	0.261
Fetal sex	0.24	0.092	-0.01	0.946
Sleep time	-0.06	0.723	-0.29	0.043*
ΔR^2^ for step 1	0.173	0.100	0.219	0.021*
*Step 2*:				
Average calories (kcal/day)	-0.14	0.317	0.23	0.076
%TDEI 12:00–15:00 h	-0.32	0.034*		
%TDEI 19:00–06:00 h			0.26	0.042*
ΔR^2^ for step 2	0.084	0.088	0.097	0.036*
F_model_	2.233	0.049*	3.305	0.006**

Abbreviation: AUC_G_ = area under the curve with respect to ground; AUC_I_ = area under the curve with respect to increase; β = standard coefficients; BMI = body mass index; TDEI = total daily energy intake. **p < 0.01; *p < 0.05

Results on the association between maternal melatonin parameters and temporal macronutrient intake were shown in Table 4. Higher percent carbohydrate (β=-0.37, p = 0.003), protein (β=-0.27, p = 0.036), and fat (β=-0.32, p = 0.034) intake during 12:00–15:59 h were associated with a lower melatonin AUC_G_. Each of them significantly predicted an additional 14.5%, 9.4%, and 9.8% of the variance. Additionally, higher fat intake in the early afternoon also significantly predicted a lower mean melatonin level (β=-0.28, p = 0.041), explaining an additional 7.5% of the variance. This indicated that higher macronutrient intake during this period significantly reduced the overall output of melatonin (mean, AUC_G_ and AUC_I_). These associations were not observed during the third trimester. As pregnancy progressed from the second to the third trimester, decreased or flatter melatonin AUC_I_ was associated with reduced percent carbohydrate intake in the early afternoon (β=-0.40, p = 0.026), accounting for an additional 14.4% of the variance. Results of the insignificant model of temporal energy and macronutrient intake were summarized in Additional File 2.

**Table 4:** Hierarchical linear regression models predicting maternal melatonin parameters from temporal macronutrient intake (n = 70)

	Melatonin parameters during the second trimester								ΔMelatonin rhythm	
	Mean		AUC_G_						ΔAUC_I_	
	β	*p*-value	β	*p*-value	β	*p*-value	β	*p*-value	β	*p*-value
*Step 1*:										
Maternal age	0.41	0.006**	0.28	0.030*	0.33	0.014*	0.42	0.003**	0.23	0.183
Pre-pregnancy BMI	-0.10	0.456	-0.33	0.010*	-0.21	0.085	-0.25	0.057	-0.22	0.178
Gestational age	0.07	0.578	-0.14	0.270	-0.20	0.097	-0.11	0.398	-0.51	0.006**
Fetal sex	0.15	0.289	-0.01	0.910	0.05	0.701	0.03	0.817	0.001	0.996
Sleep time	-0.44	0.005**	-0.19	0.164	-0.26	0.055	-0.31	0.028*	0.07	0.683
ΔR^2^ for step 1	0.279	0.014*	0.223	0.024*	0.278	0.006**	0.311	0.005**	0.274	0.105
*Step 2*:										
*Carbohydrate*:										
Average carbohydrate (g/day)	-	-	0.15	0.241	-	-	-	-	-	-
% Carbohydrate 12:00–15:00 h	-	-	-0.37	0.003**	-	-	-	-	-	-
ΔAverage carbohydrate (g/day)	-	-	-	-	-	-	-	-	0.28	0.122
Δ% Carbohydrate 12:00–15:00 h	-	-	-	-	-	-	-	-	-0.40	0.026*
*Protein*:										
Average protein (g/day)	-	-	-	-	0.25	0.049*	-	-	-	-
% Protein 12:00–15:00 h	-	-	-	-	-0.27	0.036*	-	-	-	-
*Fat*:										
Average fat (g/day)	-0.03	0.821	-	-	-	-	0.10	0.421	-	-
% Fat 12:00–15:00 h	-0.28	0.041*	-	-	-	-	-0.32	0.014*	-	-
ΔR^2^ for step 2	0.075	0.110	0.145	0.007**	0.094	0.037*	0.098	0.040*	0.144	0.063
F_model_	3.135	0.010*	3.995	0.002**	3.987	0.002**	4.148	0.002**	2.561	0.039*

Abbreviations: AUC_G_ = area under the curve with respect to ground; AUC_I_ = area under the curve with respect to increase; β = standard coefficients; BMI = body mass index; Δ = difference between the second and the third trimester. **p < 0.01; *p < 0.05

## Discussion

The findings of this study add to the limited body of research on maternal melatonin levels during pregnancy and its association with temporal energy and macronutrient distribution. Furthermore, the current findings on maternal melatonin levels in healthy pregnancies extend previous knowledge about the variation in maternal melatonin levels across gestation. Results from the present study showed that both the melatonin level and output across the day were largely maintained as pregnancy advanced. Pregnant women showed delayed temporal energy distribution with a circadian variation in macronutrient intake across the day. Higher mid-day energy and macronutrient intakes were associated with reduced melatonin levels and output. On the other hand, higher energy intake at night was associated with an overall increase in melatonin secretion over the day.

In this study, pregnant women displayed a distinct variation in melatonin levels across the day, with the lowest level in the afternoon and the highest level in the middle of the night. Pregnant women in the present study did not display a significant rise in melatonin levels as pregnancy progressed. This is contrary to previous studies that reported a 1.4 to 3-fold increment in serum melatonin concentrations in the third trimester [43, 49–51]. The overall rise in maternal melatonin levels as pregnany advances may serve an important role in preparing the mother’s body for labour induction [52].

The distinct pattern of melatonin secretion is consistent with previous studies that reported a rhythmic secretion of melatonin during gestation [49, 53]. This distinct pattern of melatonin secretion, except for amplitude was maintained across the second and third trimesters. The mean salivary melatonin levels in this study were around 2.5 to 10 times lower than previously reported mean serum and salivary melatonin levels in pregnant women [49, 50, 53]. This can be due to methodological differences including gestational week at the time of sampling, sampling method and conditions, and time and frequency of sampling. Sampling time and frequency matter due to the rhythmic nature of melatonin secretion throughout the 24 h day [54]. The concentration of melatonin in serum is normally 3-times higher than in saliva, which only reflects the level of unbound melatonin [55]. Although the pregnant women in this study were instructed to collect their 21:00 and 3:00 h salivary samples in the dark or under minimal dim light, non-compliance, where sampling was done under typical home artificial lighting, may still be present. Artificial light at night (ALAN) can have a suppressing effect on melatonin secretion [56]. As this study did not examine ALAN exposure among pregnant women, it is uncertain the degree to which ALAN can explain the variations in the melatonin level of pregnant women at 3:00 h. Hence, it is important to take into account the limitations in the sampling methodology when comparing the melatonin levels observed in this study. Furthermore, geographic differences may contribute to the disparities across studies. As the master clock of the circadian system is primarily entrained by the light/dark cycle, the population’s circadian rhythm can differ by geographical location due to variations in sunrise and sunset time. The present study is conducted in Malaysia, which is located close to the equator with approximately 12 h of daylight throughout the year [40].

The current findings showed a delayed temporal energy intake among the pregnant women whereby a higher proportion of energy was consumed in the afternoon and at night than in the morning. This observation concurs with the previous studies that similarly reported a higher prevalence of energy intake during the later times of the day or at night [57–59]. This temporal energy distribution may be related to the circadian nature of hunger and appetite which regulate energy intake. The circadian rhythm of hunger is characterized by a trough in the biological morning and the peak occurring during the biological night [60, 61]. This may explain the consistency in the findings on delayed temporal energy distribution reported by several studies on different study populations of various geographical locations. Second, higher energy consumption in the earlier times of the day has been shown to promote satiety and hence, reduce energy consumption at night [59, 62]. With a relatively lower energy intake in the morning in proportion to the rest of the day, it is possible that one may consume more energy at later times of the day. Delayed temporal energy intake is not specific to the pregnant population but to adults in general. Increased energy intake later in the day has been reported to be a global trend due to rapid urbanization and the widespread use of artificial lighting that favours a later sleep/wake timing and around-the-clock dining that may be more prevalent in the current study sample that is made up of pregnant women living in the urban city [63, 64].

The pattern in which macronutrients are ingested also appeared to vary across the day. A higher proportion of carbohydrates was consumed in the morning, more protein was consumed in the early afternoon, whereas the evening food intake was higher in fat. This is consistent with the previous studies that showed a similar circadian pattern in macronutrient intake [59, 65]. There are underlying physiological factors that drive the circadian variation in macronutrient consumption. The oxidation of utilization of carbohydrates and lipids followed a circadian pattern, where carbohydrate oxidation peaks in the biological morning and reaches the lowest value in the biological evening [66]. In contrast, lipid oxidation was highest in the biological evening and lowest in the biological morning [28, 66–68].

The circadian variation in macronutrient intake is also reflected in the local food consumption patterns in Malaysia. For instance, ‘*nasi lemak*’ or rice dish cooked in coconut milk and pandan leaf, served with fried anchovies, peanut, hard-boiled eggs, slices of cucumber, and *Sambal* is commonly consumed by Malaysians as breakfast [69]. Other than that, carbohydrate-rich foods such as roti canai, chapati, bread, rice, and noodles are also commonly eaten as breakfast in Malaysia [70, 71]. The composition of lunch and dinner is usually balanced with macronutrients, which consist of carbohydrate-rich food, vegetables and protein (Lipoeto et al. 2013). Taken together, circadian variation in macronutrient oxidation and cultural factors may explain the circadian variation in macronutrient intake in this study. Due to the lack of studies examining temporal macronutrient intake among the pregnant population, it remains uncertain whether the temporal macronutrient intake observed in the present study is contributed by pregnancy-specific factors.

The majority of the significant associations between melatonin parameters particularly mean, AUC_G_, and AUC_I_ and temporal intake of energy and macronutrients were detected in the early afternoon from 12:00 to 15:59 h. The early afternoon period is typically when the nadir of circadian melatonin rhythm occurs. This is supported by the observation in the present study that the melatonin concentration at 15:00 h was the lowest as compared to that measured at 9:00, 21:00, and 3:00 h. The underlying mechanism behind the role of mid-day energy intake on melatonin secretion may involve the interactions between melatonin and metabolic hormones, considering that this period is a metabolically active phase. Other than its role in sleep promotion, melatonin is also involved in mediating the circadian regulation of metabolic hormones such as insulin [72–74]. Melatonin also acts to maintain the internal circadian synchronization between feeding/fasting rhythm and the metabolic processes by preparing and altering the peripheral metabolic tissues to respond to the metabolic hormones secreted in response to the feeding/fasting cues [74–76]. Hence, it is plausible that the temporal pattern by which energy is consumed may influence the rhythms of the feeding/fasting rhythm and metabolic hormones, subsequently altering melatonin secretion in response to the circadian signals produced by the peripheral clocks. This may explain the association between the higher energy intake during the early afternoon period and flatter melatonin AUC_I_, which indicates an attenuated melatonin increment in the evening. Besides, results from the current study also showed trimester-specific associations, which may be due to physiological changes at different trimesters of pregnancy.

It is noteworthy that maternal melatonin levels also appeared to be sensitive to dietary manipulations at the level of temporal macronutrient intake. In this study, higher carbohydrate, protein, and fat intake during 12:00–15:59 h predicted a reduced output of melatonin as measured by mean level and AUC_G_. Previous studies have demonstrated the circadian entrainment effect of high carbohydrate and fat intake on circadian rhythm, particularly at the peripheral level. For instance, high-fat diet has been shown to increase the period length of the circadian peripheral clock in animal studies [77, 78]. Another clinical study found that switching from a high carbohydrate/low-fat diet to a high fat/low carbohydrate diet resulted in a phase delay in cortisol rhythm and altered peripheral gene expression [79]. On the other hand, temporal intake of carbohydrates is also able to alter the circadian rhythm. Restricting carbohydrate consumption to the evening while controlling the energy intake throughout the day was shown to alter the rhythms of leptin and ghrelin secretion [80]. Meanwhile, a high protein meal-induced rise in cortisol, a widely studied circadian marker, was also observed to be time-dependent [81]. Findings from this study extend the evidence emphasizing that time-of-the-day variation in macronutrient intake also plays a role in influencing the output of melatonin secretion. It is stipulated that mid-day macronutrient intake may exert a similar influence on energy intake in altering the regulation of metabolic pathways in peripheral tissues. With the circadian variation in glucose tolerance, insulin sensitivity, leptin, ghrelin, carbohydrate oxidation, and lipid oxidation, it is plausible that temporal energy and macronutrient intakes can vary the circadian response of metabolic hormones, resulting in altered melatonin secretion over the day.

The current findings also showed that higher energy intake at night (19:00 to 06:00 h) was associated with a higher melatonin AUC_G_ or a greater overall output of melatonin secretion across the day. Melatonin production normally takes place during the biological night and reaches its peak in the middle of the night [53]. The rest/sleep phase of the day or the biological night is the usual fasting period, characterized by insulin resistance, low glucose tolerance, adipose tissue lipolysis, leptin secretion, and the onset of melatonin [75]. Given that nighttime is considered a biologically resting phase, higher energy intake during this period may interrupt the circadian organization of metabolic processes and subsequently affect the output of melatonin secretion. Past evidence shows that patients with night eating syndrome (NES) displayed a delayed circadian phase and reduced amplitude of leptin, insulin, melatonin, and cortisol, with a reversed glucose rhythm [26]. A study showed that participants with NES displayed a lower nocturnal melatonin level than the control group [82]. On the other hand, a more recent study found no significant difference in melatonin levels between NES participants and control groups but showed significant differences in the levels of glucose, insulin, and ghrelin [83]. However, these findings cannot be compared directly as NES is an eating disorder that is characterized by morning anorexia, evening overeating, and insomnia [82]. Nonetheless, the findings from the current study suggest that consumption of a high amount of energy during nighttime has an influence on melatonin secretion. As the present study only examined melatonin levels at four time points across the 24 h day, it remains uncertain whether the association between higher energy intake at night (19:00 to 06:00 h) and a higher melatonin AUC_G_ or total output is contributed by the disruption in circadian melatonin secretion.

The findings should be interpreted in light of the limitations of this study. The relatively small sample size may have reduced statistical power and led to null findings. Replication is needed in larger samples. Four measurements of melatonin concentration in a day may not fully capture the rise in melatonin concentration, particularly during the nighttime. This may lead to the underestimation of the output of melatonin secretion across the day. This method also does not allow the full characterization of the melatonin rhythm. Artificial light exposure prior to night sampling may differ between the participants and can potentially contribute to suppressed melatonin secretion. This may influence melatonin concentration measured at 21:00 h and lead to underestimation. Other potential covariates of circadian melatonin secretion, such as psychological state, sleep quality and physical activity which may explain the association between temporal dietary intake and melatonin rhythm were not assessed in this study. Despite these limitations, an important strength of this study is the multiple salivary sampling across a 24 h day for a full characterization of the changes in melatonin levels across the day and night. Evaluation of maternal melatonin profile during the second and third trimesters provided comprehensive information on the change in maternal melatonin levels as pregnancy progressed.

## Conclusion

Findings of the present study provide valuable information about the trimester-specific association between maternal melatonin levels and temporal dietary intake. These results suggest that temporal energy and macronutrient intake, particularly in the early afternoon and at night, may have a more significant influence on the output and levels of circadian melatonin secretion. Our findings emphasize the role of the temporal aspect of dietary intake as a potential entrainment factor of melatonin secretion, which may have a greater metabolic and physiologic importance. Future studies with a larger sample size are warranted to replicate these findings. Understanding the mechanism underlying the association between melatonin secretion and chrononutrition may allow better management of maternal health during pregnancy through time-based dietary interventions.

## Electronic supplementary material

Below is the link to the electronic supplementary material.

Supplementary Material 1

Supplementary Material 2

## Data Availability

All data generated or analysed during this study are included in this published article and its supplementary information file.

## Abbreviations

BMI: body mass index
RM: Ringgit Malaysia
T2: second trimester
T3: third trimester.

## References

[1] Serón-Ferré M, Valenzuela GJ, Torres-Farfan C (2007). Circadian clocks during embryonic and fetal development. Birth Defects Res C Embryo Today.

[2] Tan D, Reiter R, Manchester L, Yan M, El-Sawi M, Sainz R (2002). Chemical and Physical Properties and potential mechanisms: melatonin as a broad spectrum antioxidant and free radical scavenger. Curr Top Med Chem.

[3] Santhi N, Thorne HC, van der Veen DR, Johnsen S, Mills SL, Hommes V (2012). The spectral composition of evening light and individual differences in the suppression of melatonin and delay of sleep in humans. J Pineal Res.

[4] Cardinali DP, Srinivasan V, Brzezinski A, Brown GM (2012). Melatonin and its analogs in insomnia and depression. J Pineal Res.

[5] Torres-Farfan C, Rocco V, Monsó C, Valenzuela FJ, Campino C, Germain A (2006). Maternal melatonin effects on clock gene expression in a nonhuman primate fetus. Endocrinology.

[6] Gozeri E, Celik H, Ozercan I, Gurates B, Polat SA, Hanay F (2008). The effect of circadian rhythm changes on fetal and placental development (experimental study). Neuro Endocrinol Lett.

[7] Summa KC, Vitaterna MH, Turek FW (2012). Environmental perturbation of the circadian clock disrupts pregnancy in the mouse. PLoS ONE.

[8] Gatford KL, Kennaway DJ, Liu H, Kleemann DO, Kuchel TR, Varcoe TJ (2019). Simulated shift work disrupts maternal circadian rhythms and metabolism, and increases gestation length in sheep. J Physiol.

[9] Reiter RJ, Tan DX, Korkmaz A, Rosales-Corral SA (2014). Melatonin and stable circadian rhythms optimize maternal, placental and fetal physiology. Hum Reprod Update.

[10] Reppert SM, Schwartz WJ. Maternal Coordination of the Fetal Biological Clock in Utero. Science (1979) 1983;220:969–71. 10.1126/science.6844923. 10.1126/science.6844923 6844923

[11] Reppert S, Schwartz W (1984). The suprachiasmatic nuclei of the fetal rat: characterization of a functional circadian clock using 14 C-labeled deoxyglucose. J Neurosci.

[12] Jud C, Albrecht U (2006). Circadian rhythms in murine pups develop in absence of a functional maternal circadian clock. J Biol Rhythms.

[13] Wreschnig D, Dolatshad H, Davis FC (2014). Embryonic Development of Circadian Oscillations in the mouse hypothalamus. J Biol Rhythms.

[14] Mohawk JA, Green CB, Takahashi JS (2012). Central and peripheral circadian clocks in mammals. Annu Rev Neurosci.

[15] Dibner C, Schibler U, Albrecht U. The mammalian circadian timing system: Organization and coordination of central and peripheral clocks. vol. 72. 2009. 10.1146/annurev-physiol-021909-135821. 10.1146/annurev-physiol-021909-135821 20148687

[16] Froy O (2010). Metabolism and circadian rhythms - implications for obesity. Endocr Rev.

[17] López-Gambero AJ, Martínez F, Salazar K, Cifuentes M, Nualart F (2019). Brain glucose-sensing mechanism and energy homeostasis. Mol Neurobiol.

[18] Stokkan KA, Yamazaki S, Tei H, Sakaki Y, Menaker M. Entrainment of the circadian clock in the liver by feeding. Science (1979) 2001;291:490–3. 10.1126/science.291.5503.490. 10.1126/science.291.5503.490 11161204

[19] Feillet CA, Mendoza J, Albrecht U, Pévet P, Challet E (2008). Forebrain oscillators ticking with different clock hands. Mol Cell Neurosci.

[20] Wehrens SMT, Christou S, Isherwood C, Middleton B, Gibbs MA, Archer SN (2017). Meal timing regulates the human circadian system. Curr Biol.

[21] Bray MS, Ratcliffe WF, Grenett MH, Brewer RA, Gamble KL, Young ME (2013). Quantitative analysis of light-phase restricted feeding reveals metabolic dyssynchrony in mice. Int J Obes.

[22] Tahara Y, Shibata S (2013). Chronobiology and nutrition. Neuroscience.

[23] Witbracht M, Keim NL, Forester S, Widaman A, Laugero K (2015). Female breakfast skippers display a disrupted cortisol rhythm and elevated blood pressure. Physiol Behav.

[24] Ogata H, Horie M, Kayaba M, Tanaka Y, Ando A, Park I (2020). Skipping breakfast for 6 days delayed the circadian rhythm of the body temperature without altering clock gene expression in human leukocytes. Nutrients.

[25] Shimizu H, Hanzawa F, Kim D, Sun S, Laurent T, Umeki M (2018). Delayed first active-phase meal, a breakfastskipping model, led to increased body weight and shifted the circadian oscillation of the hepatic clock and lipid metabolism-related genes in rats fed a high-fat diet. PLoS ONE.

[26] Goel N, Stunkard AJ, Rogers NL, Van Dongen HPA, Allison KC, O’Reardon JP (2009). Circadian rhythm profiles in women with night eating syndrome. J Biol Rhythms.

[27] Ni Y, Wu L, Jiang J, Yang T, Wang Z, Ma L (2019). Late-Night Eating‐Induced physiological dysregulation and circadian misalignment are accompanied by Microbial Dysbiosis. Mol Nutr Food Res.

[28] Bandín C, Scheer FAJL, Luque AJ, Ávila-Gandiá V, Zamora S, Madrid JA (2015). Meal timing affects glucose tolerance, substrate oxidation and circadian-related variables: a randomized, crossover trial. Int J Obes.

[29] Brown RF, Thorsteinsson EB, Smithson M, Birmingham CL, Aljarallah H, Nolan C (2017). Can body temperature dysregulation explain the co-occurrence between overweight/obesity, sleep impairment, late-night eating, and a sedentary lifestyle?. Eat Weight Disorders.

[30] Kaur S, Teoh AN, Shukri NHM, Shafie SR, Bustami NA, Takahashi M (2020). Circadian rhythm and its association with birth and infant outcomes: Research protocol of a prospective cohort study. BMC Pregnancy Childbirth.

[31] Ogata H, Hatamoto Y, Goto Y, Tajiri E, Yoshimura E, Kiyono K (2019). Association between breakfast skipping and postprandial hyperglycaemia after lunch in healthy young individuals. Br J Nutr.

[32] Tee ES, Mohd Ismail N, Mohd Nasir A, Khatijah I (1997). Nutrient composition of malaysian food.

[33] United States Department of Agriculture & Agricultural Research Service (1998). Continuing Survey of Food Intakes by individuals, 1994–1996.

[34] Puwastien B, Burlingame M, Raroengwichit P (2000). ASEAN Food Composition tables.

[35] Ministry of Health Singapore (2001). National Nutrition Survey, Singapore, 1998.

[36] Mohd Ismail N, Ng K, Chee S, Roslee R, Zawiah H (1998). Predictive equations for estimation of basal metabolic rate in malaysian adults. Malays J Nutr.

[37] Goldberg G, Black A, Jebb S, Cole T, Murgatroyd P, Coward W (1991). Critical evaluation of energy intake data using fundamental principles of energy physiology: 1. Derivation of cut-off limits to identify under-recording. Eur J Clin Nutr.

[38] Black AE. Critical evaluation of energy intake using the Goldberg cut-off for energy intake:basal metabolic rate. A practical guide to its calculation, use and limitations. vol. 24. 2000. 10.1038/sj.ijo.0801376 11033980

[39] Ministry of Communications and Multimedia Malaysia. Department of Information Malaysia 2020. www.penerangan.gov.my (accessed October 2, 2021).

[40] WorldData.info. Sunrise and sunset in Malaysia 2022. https://www.worlddata.info/asia/malaysia/sunset.php (accessed October 2, 2021).

[41] Loy SL, Loo RSX, Godfrey KM, Chong YS, Shek LPC, Tan KH (2020). Chrononutrition during pregnancy: a review on maternal night-time eating. Nutrients.

[42] Zhou JN, Liu RY, Van Heerikhuize J, Hofman MA, Swaab DF (2003). Alterations in the circadian rhythm of salivary melatonin begin during middle-age. J Pineal Res.

[43] Mirdamadi K. Salivary melatonin levels in pregnant women with Insomnia: a prospective cohort study with two comparison groups. 2016. 10.13140/RG.2.1.1007.3204.

[44] Middleton B (2013). Measurement of melatonin and 6-sulphatoxymelatonin. Methods Mol Biol.

[45] Zarazaga L, Malpaux B, Chemineau P (2003). Amplitude of the plasma melatonin nycthemeral rhythms is not associated with the dates of onset and offset of the seasonal ovulatory activity in the ile-de-france ewe. Reprod Nutr Dev.

[46] Pruessner JC, Kirschbaum C, Meinlschmid G, Hellhammer DH (2003). Two formulas for computation of the area under the curve represent measures of total hormone concentration versus time-dependent change. Psychoneuroendocrinology.

[47] Kivlighan KT, DiPietro JA, Costigan KA, Laudenslager ML (2008). Diurnal rhythm of cortisol during late pregnancy: Associations with maternal psychological well-being and fetal growth. Psychoneuroendocrinology.

[48] National Coordinating Committee on Food and Nutrition. Recommended nutrient intakes for Malaysia. 2017.

[49] Kivela A (1991). Serum melatonin during human pregnancy. Acta Endocrinol (Copenh).

[50] Nakamura Y, Tamura H, Kashida S, Takayama H, Yamagata Y, Karube A (2001). Changes of serum melatonin level and its relationship to feto-placental unit during pregnancy. J Pineal Res.

[51] Ejaz H, Figaro JK, Woolner AMF, Thottakam BMV, Galley HF (2021). Maternal serum melatonin increases during pregnancy and Falls immediately after delivery implicating the Placenta as a major source of Melatonin. Front Endocrinol (Lausanne).

[52] Gögenur I, Ocak U, Altunpinar Ö, Middleton B, Skene DJ, Rosenberg J (2007). Disturbances in melatonin, cortisol and core body temperature rhythms after major surgery. World J Surg.

[53] Shimada M, Seki H, Samejima M, Hayase M, Shirai F (2016). Salivary melatonin levels and sleep-wake rhythms in pregnant women with hypertensive and glucose metabolic disorders: a prospective analysis. Biosci Trends.

[54] Rzepka-migut B, Paprocka J. Melatonin‐measurement methods and the factormodifying the results. A systematic review of thliterature. Int J Environ Res Public Health 2020;17. 10.3390/ijerph17061916. 10.3390/ijerph17061916 PMC7142625 32183489

[55] Dermanowski MM, Hejduk A, Kuczyńska J, Wichniak A, Urbańska A, Mierzejewski P. Assessment of dim light melatonin onset based on plasma and saliva samples. Chronobiol Int 2022:1–10. 10.1080/07420528.2021.2016796. 10.1080/07420528.2021.2016796 35168448

[56] Reiter RJ, Tan DX, Korkmaz A, Erren TC, Piekarski C, Tamura H (2007). Light at night, chronodisruption, melatonin suppression, and cancer risk: a review. Crit Rev Oncog.

[57] Loy SL, Cheng TS, Colega MT, Cheung YB, Godfrey KM, Gluckman PD (2016). Predominantly night-time feeding and maternal glycaemic levels during pregnancy. Br J Nutr.

[58] Chandler-Laney PC, Schneider CR, Gower BA, Granger WM, Mancuso MS, Biggio JR (2016). Association of late-night carbohydrate intake with glucose tolerance among pregnant african american women. Matern Child Nutr.

[59] de Castro JM (1987). Circadian rhythms of the spontaneous meal pattern, macronutrient intake, and mood of humans. Physiol Behav.

[60] Scheer FAJL, Morris CJ, Shea SA (2013). The internal circadian clock increases hunger and appetite in the evening independent of food intake and other behaviors. Obesity.

[61] Sargent C, Zhou X, Matthews RW, Darwent D, Roach GD. Daily rhythms of hunger and satiety in healthy men during one week of sleep restriction and circadian misalignment. Int J Environ Res Public Health. 2016;13. 10.3390/ijerph13020170. 10.3390/ijerph13020170 PMC4772190 26840322

[62] Gontijo CA, Balieiro LCT, Teixeira GP, Fahmy WM, Crispim CA, de Maia YC. P. Higher energy intake at night effects daily energy distribution and contributes to excessive weight gain during pregnancy. Nutrition 2020;74. 10.1016/j.nut.2020.110756. 10.1016/j.nut.2020.110756 32278857

[63] Almoosawi S, Vingeliene S, Karagounis LG, Pot GK (2016). Chrono-nutrition: a review of current evidence from observational studies on global trends in time-of-day of energy intake and its association with obesity. Proc Nutr Soc.

[64] Goh E, Von, Azam-Ali S, McCullough F, Roy Mitra S (2020). The nutrition transition in Malaysia; Key drivers and recommendations for improved health outcomes. BMC Nutr.

[65] McHill AW, Czeisler CA, Phillips AJK, Keating L, Barger LK, Garaulet M et al. Caloric and macronutrient intake differ with circadian phase and between lean and overweight young adults. Nutrients 2019;11. 10.3390/nu11030587. 10.3390/nu11030587 PMC6471585 30862011

[66] Zitting KM, Vujovic N, Yuan RK, Isherwood CM, Medina JE, Wang W (2018). Human resting energy expenditure varies with circadian phase. Curr Biol.

[67] McHill AW, Melanson EL, Higgins J, Connick E, Moehlman TM, Stothard ER (2014). Impact of circadian misalignment on energy metabolism during simulated nightshift work. Proc Natl Acad Sci U S A.

[68] Morris CJ, Yang JN, Garcia JI, Myers S, Bozzi I, Wang W (2015). Endogenous circadian system and circadian misalignment impact glucose tolerance via separate mechanisms in humans. Proc Natl Acad Sci U S A.

[69] Omar SR, Omar SN (2018). Malaysian Heritage Food (MHF): a review on its unique Food Culture, tradition and Present Lifestyle. Int J Herit Art Multimedia.

[70] Lipoeto NI, Geok Lin K, Angeles-Agdeppa I (2013). Food consumption patterns and nutrition transition in South-East Asia. Public Health Nutr.

[71] Mustafa N, Abd Majid H, Toumpakari Z, Carroll HA, Yazid Jalaludin M, Al Sadat N (2019). The Association of Breakfast frequency and Cardiovascular Disease (CVD) risk factors among adolescents in Malaysia. Nutrients.

[72] Cailotto C, La Fleur SE, Van Heijningen C, Wortel J, Kalsbeek A, Feenstra M (2005). The suprachiasmatic nucleus controls the daily variation of plasma glucose via the autonomic output to the liver: are the clock genes involved?. Eur J Neurosci.

[73] La Fleur SE, Kalsbeek A, Wortel J, Van Der Vliet J, Buijs RM (2001). Role for the pineal and melatonin in glucose homeostasis: Pinealectomy increases night- time glucose concentrations. J Neuroendocrinol.

[74] Cipolla-Neto J, Amaral FG, Afeche SC, Tan DX, Reiter RJ (2014). Melatonin, energy metabolism, and obesity: a review. J Pineal Res.

[75] Alonso-Vale MIC, Andreotti S, Borges-Silva CDN, Mukai PY, Cipolla-Neto J, Lima FB (2006). Intermittent and rhythmic exposure to melatonin in primary cultured adipocytes enhances the insulin and dexamethasone effects on leptin expression. J Pineal Res.

[76] Alonso-Vale MIC, Andreotti S, Peres SB, Anhê GF, Borges-Silva CDN, Neto JC, et al. Melatonin enhances leptin expression by rat adipocytes in the presence of insulin. Am J Physiol Endocrinol Metab. 2005;288. 10.1152/ajpendo.00478.2004. 10.1152/ajpendo.00478.2004 15572654

[77] Eckel-Mahan KL, Patel VR, De Mateo S, Orozco-Solis R, Ceglia NJ, Sahar S (2013). Reprogramming of the circadian clock by nutritional challenge. Cell.

[78] Branecky KL, Niswender KD, Pendergast JS (2015). Disruption of daily rhythms by high-fat diet is reversible. PLoS ONE.

[79] Pivovarova O, Jürchott K, Rudovich N, Hornemann S, Ye L, Möckel S (2015). Changes of dietary fat and carbohydrate content alter central and peripheral clock in humans. J Clin Endocrinol Metab.

[80] Sofer S, Eliraz A, Kaplan S, Voet H, Fink G, Kima T (2013). Changes in daily leptin, ghrelin and adiponectin profiles following a diet with carbohydrates eaten at dinner in obese subjects. Nutr Metabolism Cardiovasc Dis.

[81] Slag MF, Ahmed M, Gannon MC, Nuttall FQ (1981). Meal stimulation of cortisol secretion: a protein induced effect. Metabolism.

[82] Birketvedt GS (1999). Behavioral and neuroendocrine characteristics of the night-eating syndrome. JAMA.

[83] Allison KC, Ahima RS, O’Reardon JP, Dinges DF, Sharma V, Cummings DE (2005). Neuroendocrine Profiles Associated with Energy Intake, Sleep, and stress in the Night Eating Syndrome. J Clin Endocrinol Metab.

