# Supplementary figures and images for: Maternal melatonin levels and temporal dietary intake: results from MY-CARE cohort study
Source: BMC Pregnancy Childbirth. 2023 Jul 4;23:491. doi: 10.1186/s12884-023-05796-y (PMC10318628; doi:10.1186/s12884-023-05796-y)

**
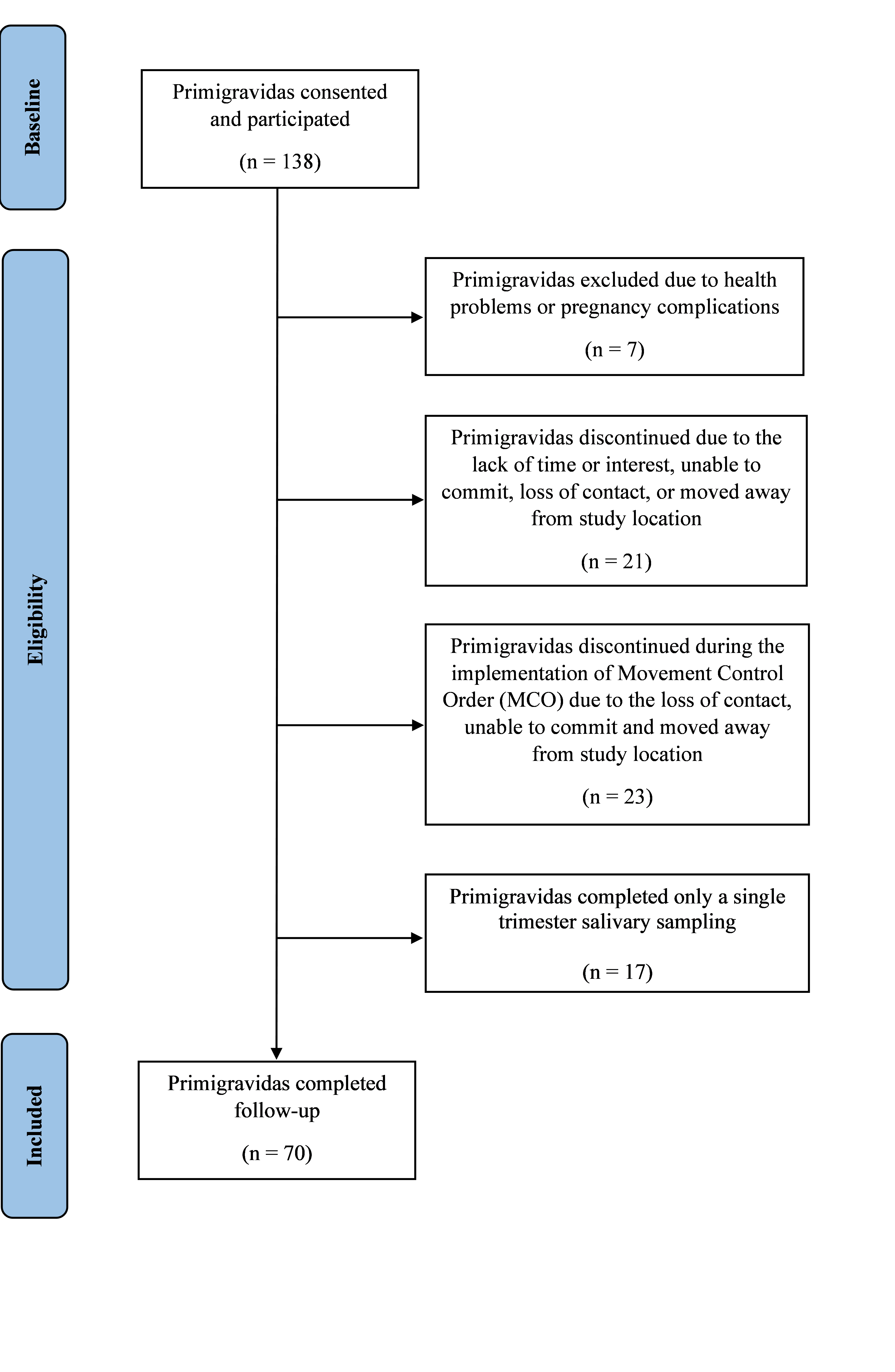
**

**Figure S1.** Flowchart of recruitment and participation of pregnant women in this study.

Supplement: Supplementary file 1 — Supplementary Material 1 [file 12884_2023_5796_MOESM1_ESM.docx]
