# Supplementary material for: Maternal melatonin levels and temporal dietary intake: results from MY-CARE cohort study
Source: BMC Pregnancy Childbirth. 2023 Jul 4;23:491. doi: 10.1186/s12884-023-05796-y (PMC10318628; doi:10.1186/s12884-023-05796-y)
Supplement: Supplementary file 2 — Supplementary Material 2 [file 12884_2023_5796_MOESM2_ESM.docx]

**Table S1.** Salivary melatonin levels of the study samples (n=70).

|  | **T2** | **T3** | ***p*-value** |
| --- | --- | --- | --- |
|  | **Mean ± SD/ Median (IQR)** | |  |
| Average wake time (hh:mm) | 7:46 ± 1:13 | 7:52 ± 1:11 | 0.375 |
| Average sleep time (hh:mm) | 23:40 ± 1:07 | 24:03 ± 1:09 | 0.011* |
| Melatonin at 09:00 (pmol/l) | 6.34 (3.42, 6.34) | 7.35 (3.41, 12.94) | 0.379 |
| Melatonin at 15:00 (pmol/l) | 3.09 (1.40, 4.86) | 3.07 (1.19, 5.77) | 0.215 |
| Melatonin at 21:00 (pmol/l) | 5.62 (2.33, 11.47) | 4.20 (1.60, 8.73) | 0.420 |
| Melatonin at 03:00 (pmol/l) | 26.68 (10.90, 44.72) | 20.27 (6.06, 43.83) | 0.695 |
| Mean (pmol/l) | 12.74 (8.85, 19.95) | 11.89 (4.27, 19.84) | 0.161 |
| Amplitude | 31.19 (12.5, 46.45) | 12.62 (5.38, 27.57) | 0.020* |
| Maximal level (pmol/l/) | 31.30 (17.58, 48.84) | 22.24 (9.73, 44.24) | 0.719 |
| AUC_G_ (pmol/l) | 33.90 (23.04, 53.32) | 28.32 (10.51, 45.38) | 0.087 |
| AUC_I_ (pmol/l/h) | 12.94 (-5.59, 32.47) | 6.43 (-1.23, 28.99) | 0.586 |

Abbreviations: AUC_G_ = area under the curve with respect to ground; AUC_I_ = area under the curve with respect to increase; IQR = interquartile range; SD = standard deviation; T2 = second trimester; T3 = third trimester.

**p*<0.05.

**Table S2.** Temporal intake of energy and macronutrient across the 24h day among the study samples (n=70).

| **Nutrient intake** | **T2 (n = 64)** | | **T3 (n = 60)** | |
| --- | --- | --- | --- | --- |
|  | **Mean** | **SD** | **Mean** | **SD** |
| **Energy (kcal/d)** |  |  |  |  |
| 7:00 to 11:59h | 617.81 | 242.25 | 584.27 | 207.57 |
| 12:00 to 15:59h | 646.65 | 226.74 | 627.23 | 140.92 |
| 16:00 to 18:59h, median | 276.47 | 78.59, 431.94 | 195.29 | 48.43, 348.06 |
| 19:00 to 23:59h | 627.65 | 206.46 | 682.64 | 194.43 |
| 24:00 to 6:00h, median | 0.00 | 0.00, 0.00 | 0.00 | 0.00, 0.00 |
| **Carbohydrate (g/d)** |  |  |  |  |
| 7:00 to 11:59h | 80.11 | 36.26 | 74.28 | 32.66 |
| 12:00 to 15:59h | 76.01 | 23.18 | 76.49 | 30.12 |
| 16:00 to 18:59h, median | 26.35 | 3.91, 60.34 | 21.63 | 2.21, 45.48 |
| 19:00 to 23:59h | 72.24 | 25.56 | 72.02 | 31.27 |
| 24:00 to 6:00h, median | 0.00 | 0.00, 0.00 | 0.00 | 0.00, 0.00 |
| **Protein (g/d)** |  |  |  |  |
| 7:00 to 11:59h | 18.87 | 7.63 | 21.04 | 12.32 |
| 12:00 to 15:59h | 30.59 | 10.43 | 30.13 | 10.99 |
| 16:00 to 18:59h, median | 4.13 | 10 | 3.84 | 0.12, 9.52 |
| 19:00 to 23:59h | 28.48 | 9.85 | 31.35 | 12.25 |
| 24:00 to 6:00h, median | 0.00 | 0.00, 0.00 | 0.00 | 0.00, 0.00 |
| **Fat (g/d)** |  |  |  |  |
| 7:00 to 11:59h | 21.71 | 9.65 | 22.39 | 11.15 |
| 12:00 to 15:59h | 25.64 | 8.07 | 26.05 | 10.13 |
| 16:00 to 18:59h, median | 7.28 | 0.48, 16.55 | 7.67 | 0.32, 16.50 |
| 19:00 to 23:59h | 25.69 | 9.94 | 25.35 | 11.09 |
| 24:00 to 6:00h, median | 0.00 | 0.00, 0.00 | 0.00 | 0.00, 0.00 |

Abbreviations: SD = standard deviation; T2 = second trimester; T3 = third trimester.

*p<0.05; **p<0.01.

**Table S3.** Results of hierarchical linear regression models on maternal melatonin parameters and temporal energy intake.

|  | **Mean** | | **Amplitude** | | **Maximal level** | | **AUC_G_** | | **AUC_I_** | |
| --- | --- | --- | --- | --- | --- | --- | --- | --- | --- | --- |
|  | β | *p*-value | β | *p*-value | β | *p*-value | β | *p*-value | β | *p*-value |
| **% TDEI 7:00-11:59h** | | | | | | | | | | |
| Second trimester | 0.03 | 0.867 | -0.06 | 0.756 | -0.11 | 0.561 | -0.12 | 0.424 | 0.26 | 0.147 |
| Third trimester | -0.24 | 0.189 | -0.05 | 0.820 | -0.26 | 0.187 | -0.22 | 0.222 | -0.13 | 0.443 |
| ΔAcross trimesters | 0.06 | 0.737 | -0.10 | 0.623 | -0.10 | 0.611 | -0.07 | 0.712 | 0.27 | 0.182 |
| **% TDEI 12:00-15:59h** | | | | | | | | | | |
| Second trimester | -0.25 | 0.090 | -0.08 | 0.631 | -0.14 | 0.375 | -0.21 | 0.119 | -0.32 | 0.034* |
| Third trimester | -0.02 | 0.890 | 0.13 | 0.548 | -0.10 | 0.570 | -0.01 | 0.952 | 0.21 | 0.196 |
| ΔAcross trimesters | -0.10 | 0.574 | 0.18 | 0.400 | -0.02 | 0.934 | -0.10 | 0.563 | -0.28 | 0.122 |
| **% TDEI 16:00-18:59h** | | | | | | | | | | |
| Second trimester | 0.03 | 0.824 | 0.1 | 0.547 | 0.07 | 0.662 | 0.01 | 0.965 | -0.02 | 0.866 |
| Third trimester | 0.06 | 0.708 | -0.19 | 0.423 | 0.003 | 0.986 | 0.03 | 0.880 | 0.20 | 0.233 |
| ΔAcross trimesters | 0.003 | 0.987 | -0.09 | 0.646 | -0.10 | 0.941 | -0.10 | 0.555 | 0.04 | 0.832 |
| **% TDEI 19:00-6:59h** | | | | | | | | | | |
| Second trimester | 0.12 | 0.417 | -0.16 | 0.308 | -0.07 | 0.636 | 0.26 | 0.042* | 0.09 | 0.535 |
| Third trimester | 0.02 | 0.898 | -0.15 | 0.438 | -0.03 | 0.863 | 0.03 | 0.876 | -0.04 | 0.787 |
| ΔAcross trimesters | 0.09 | 0.637 | 0.15 | 0.479 | 0.13 | 0.493 | 0.25 | 0.154 | -0.17 | 0.397 |

Abbreviations: AUC_G_ = area under the curve with respect to ground; AUC_I_ = area under the curve with respect to increase; β = standard coefficients; Δ = difference between the second and the third trimester; TDEI = total daily energy intake. ***p*<0.01; **p*<0.05.

**Table S4.** Results of hierarchical linear regression models on maternal melatonin parameters and temporal macronutrient intake.

|  | **Mean** | | **Amplitude** | | **Maximal level** | | **AUC_G_** | | **AUC_I_** | |
| --- | --- | --- | --- | --- | --- | --- | --- | --- | --- | --- |
|  | β | *p*-value | β | *p*-value | β | *p*-value | β | *p*-value | β | *p*-value |
| **% Carbohydrate 7:00-11:59h** | | | | | | | | | | |
| Second trimester | 0.08 | 0.646 | -0.09 | 0.636 | -0.12 | 0.530 | -0.13 | 0.455 | 0.17 | 0.357 |
| Third trimester | -0.22 | 0.153 | -0.06 | 0.784 | -0.10 | 0.527 | -0.13 | 0.426 | -0.08 | 0.595 |
| ΔAcross trimesters | 0.16 | 0.382 | 0.13 | 0.559 | 0.15 | 0.444 | 0.25 | 0.144 | 0.11 | 0.545 |
| **% Carbohydrate 12:00-15:59h** | | | | | | | | | | |
| Second trimester | -0.25 | 0.059 | -0.07 | 0.611 | -0.13 | 0.372 | -0.37 | 0.003** | -0.08 | 0.564 |
| Third trimester | 0.03 | 0.870 | 0.15 | 0.466 | 0.02 | 0.896 | 0.01 | 0.936 | 0.20 | 0.197 |
| ΔAcross trimesters | -0.11 | 0.567 | -0.04 | 0.838 | -0.26 | 0.173 | -0.09 | 0.617 | -0.40 | 0.026* |
| **% Carbohydrate 16:00-18:59h** | | | | | | | | | | |
| Second trimester | -0.25 | 0.059 | -0.07 | 0.611 | -0.13 | 0.372 | 0.03 | 0.854 | -0.08 | 0.564 |
| Third trimester | 0.03 | 0.870 | 0.15 | 0.466 | 0.02 | 0.896 | 0.01 | 0.936 | 0.20 | 0.197 |
| ΔAcross trimesters | -0.11 | 0.567 | -0.04 | 0.838 | -0.26 | 0.173 | -0.09 | 0.617 | 0.08 | 0.664 |
| **% Carbohydrate 19:00-6:59h** | | | | | | | | | | |
| Second trimester | 0.14 | 0.323 | 0.12 | 0.434 | 0.16 | 0.286 | 0.26 | 0.052 | 0.16 | 0.267 |
| Third trimester | -0.13 | 0.490 | -0.25 | 0.254 | -0.21 | 0.256 | -0.26 | 0.153 | -0.13 | 0.491 |
| ΔAcross trimesters | -0.11 | 0.535 | -0.10 | 0.622 | -0.13 | 0.470 | -0.05 | 0.776 | 0.07 | 0.696 |
| **% Protein 7:00-11:59h** | | | | | | | | | | |
| Second trimester | -0.03 | 0.853 | -0.15 | 0.371 | -0.16 | 0.330 | 0.08 | 0.602 | 0.13 | 0.463 |
| Third trimester | -0.07 | 0.680 | -0.14 | 0.499 | -0.10 | 0.558 | -0.01 | 0.950 | -0.17 | 0.293 |
| ΔAcross trimesters | 0.01 | 0.955 | 0.06 | 0.777 | -0.05 | 0.788 | 0.07 | 0.707 | -0.02 | 0.913 |
| **% Protein 12:00-15:59h** | | | | | | | | | | |
| Second trimester | -0.16 | 0.262 | 0.07 | 0.632 | 0.03 | 0.811 | -0.27 | 0.036* | -0.12 | 0.415 |
| Third trimester | 0.16 | 0.365 | -0.10 | 0.666 | 0.16 | 0.381 | 0.15 | 0.405 | 0.08 | 0.621 |
| ΔAcross trimesters | -0.03 | 0.883 | -0.003 | 0.987 | 0.10 | 0.633 | 0.003 | 0.989 | -0.08 | 0.682 |
| **% Protein 16:00-18:59h** | | | | | | | | | | |
| Second trimester | -0.11 | 0.433 | -0.08 | 0.624 | -0.14 | 0.347 | -0.10 | 0.429 | -0.07 | 0.662 |
| Third trimester | -0.11 | 0.480 | 0.28 | 0.157 | -0.04 | 0.797 | -0.10 | 0.521 | 0.07 | 0.644 |
| ΔAcross trimesters | -0.08 | 0.661 | -0.07 | 0.725 | -0.15 | 0.386 | -0.16 | 0.352 | -0.05 | 0.786 |

Abbreviations: AUC_G_ = area under the curve with respect to ground; AUC_I_ = area under the curve with respect to increase; β = standard coefficients; Δ = difference between the second and the third trimester. ***p*<0.01; **p*<0.05.

**Table S4.** *Continued.*

|  | **Mean** | | **Amplitude** | | **Maximal level** | | **AUC_G_** | | **AUC_I_** | |
| --- | --- | --- | --- | --- | --- | --- | --- | --- | --- | --- |
|  | β | *p*-value | β | *p*-value | β | *p*-value | β | *p*-value | β | *p*-value |
| **% Protein 19:00-6:59h** | | | | | | | | | | |
| Second trimester | 0.22 | 0.131 | 0.22 | 0.140 | 0.29 | 0.047* | 0.20 | 0.134 | 0.18 | 0.229 |
| Third trimester | 0.03 | 0.859 | -0.19 | 0.354 | -0.02 | 0.896 | -0.14 | 0.422 | -0.03 | 0.853 |
| ΔAcross trimesters | 0.24 | 0.197 | 0.25 | 0.223 | 0.30 | 0.090 | 0.12 | 0.517 | 0.05 | 0.769 |
| **% Fat 7:00-11:59h** | | | | | | | | | | |
| Second trimester | -0.10 | 0.573 | -0.06 | 0.752 | -0.15 | 0.433 | 0.002 | 0.988 | 0.25 | 0.154 |
| Third trimester | -0.30 | 0.155 | -0.08 | 0.731 | -0.23 | 0.300 | -0.27 | 0.203 | -0.17 | 0.382 |
| ΔAcross trimesters | 0.09 | 0.655 | -0.24 | 0.324 | -0.09 | 0.656 | -0.12 | 0.538 | -0.11 | 0.571 |
| **% Fat 12:00-15:59h** | | | | | | | | | | |
| Second trimester | -0.28 | 0.041* | -0.15 | 0.344 | -0.16 | 0.299 | -0.32 | 0.014* | -0.15 | 0.365 |
| Third trimester | 0.22 | 0.169 | -0.13 | 0.486 | 0.14 | 0.377 | 0.24 | 0.137 | 0.01 | 0.933 |
| ΔAcross trimesters | -0.01 | 0.967 | -0.01 | 0.960 | 0.02 | 0.928 | -0.09 | 0.639 | -0.25 | 0.183 |
| **% Fat 16:00-18:59h** | | | | | | | | | | |
| Second trimester | -0.06 | 0.690 | -0.01 | 0.956 | -0.03 | 0.866 | -0.01 | 0.916 | -0.03 | 0.851 |
| Third trimester | 0.15 | 0.342 | -0.14 | 0.480 | 0.13 | 0.457 | 0.07 | 0.697 | 0.08 | 0.631 |
| ΔAcross trimesters | 0.18 | 0.321 | -0.09 | 0.698 | 0.12 | 0.541 | 0.05 | 0.779 | 0.06 | 0.796 |
| **% Fat 19:00-6:59h** | | | | | | | | | | |
| Second trimester | 0.06 | 0.669 | -0.06 | 0.714 | 0.004 | 0.977 | 0.18 | 0.169 | 0.01 | 0.921 |
| Third trimester | 0.10 | 0.574 | -0.20 | 0.350 | 0.04 | 0.813 | -0.03 | 0.867 | -0.18 | 0.303 |
| ΔAcross trimesters | -0.14 | 0.468 | -0.30 | 0.181 | -0.18 | 0.359 | -0.20 | 0.283 | -0.19 | 0.325 |

Abbreviations: AUC_G_ = area under the curve with respect to ground; AUC_I_ = area under the curve with respect to increase; β = standard coefficients; Δ = difference between the second and the third trimester. ***p*<0.01; **p*<0.05.
